# The Impact of Trauma System Implementation on Patient Quality of Life and Economic Burden: A Systematic Review Study Protocol

**DOI:** 10.29337/ijsp.187

**Published:** 2023-02-09

**Authors:** Michael F. Bath, Katharina Kohler, Laura Hobbs, Isla Kuhn, William M. Nabulyato, Arthur Kwizera, Laura E. Walker, Tom Wilkins, Daniel Stubbs, Sara Halimah, Rowan Burnstein, Angelos G. Kolias, Peter Hutchinson, John Clarkson, Tom Bashford

**Affiliations:** 1 NIHR Global Health Research Group on Acquired Brain and Spine Injury, Department of Engineering & Division of Anaesthesia, University of Cambridge, Cambridge, UK; 2 World Health Organisation Trauma Operational and Advisory team, UK; 3 University of Cambridge Medical Library, University of Cambridge, Cambridge, UK; 4 Department of Anaesthesia and Intensive Care, Makerere University College of Health Sciences, Kampala, Uganda; 5 Department of Emergency Medicine, Mayo Clinic, Rochester, Minnesota, USA; 6 School of Clinical Medicine, University of Cambridge, Cambridge, UK; 7 Department of Perioperative, Acute, Critical Care, and Emergency Medicine, Department of Medicine, University of Cambridge, UK; 8 World Health Organisation Trauma Operational and Advisory team, UK; 9 NIHR Global Health Research Group on Acquired Brain and Spine Injury, Division of Academic Neurosurgery, University of Cambridge, Cambridge, UK

**Keywords:** Trauma Systems, Morbidity, Quality of Life, Economic Burden

## Abstract

**Background:**

Trauma accounts for 10% of global mortality, with increasing rates disproportionally affecting low- and middle-income countries. In an attempt to improve clinical outcomes after injury, trauma systems have been implemented in multiple countries over recent years. However, whilst many studies have subsequently demonstrated improvements in overall mortality outcomes, less is known about the impact trauma systems have on morbidity, quality of life, and economic burden. This systematic review seeks to assess the existing evidence base for trauma systems with these outcome measures.

**Methods:**

This review will include any study that assesses the impact implementation of a trauma system has on patient morbidity, quality of life, or economic burden. Any comparator study, including cohort, case-control, and randomised controlled studies, will be included, both retrospective or prospective in nature. Studies conducted from any region in the world and involving any age of patient will be included. We will collect data on any morbidity outcomes, health-related quality of life measures, or health economic assessments reported. We predict a high heterogeneity in these outcomes used and will therefore keep inclusion criteria broad.

**Discussion:**

Previous reviews have shown the significant improvements that can be achieved in mortality outcomes with the implementation of an organised trauma system, however the wider impact they can have on morbidity outcomes, quality of life measures, and the economic burden of trauma, is less well described. This systematic review will present all available data on these outcomes, helping to better characterise both the societal and economic impact of trauma system implementation.

**Highlights:**

- Trauma systems are known to improve mortality rates, however less in known on the impact they have on morbidity outcomes, quality of life, and economic burden
- We aim to perform a systematic review to identify any comparator study that assesses the impact implementation of a trauma system on these outcomes
- Understanding the impact trauma systems can have on wider parameters, such as economic and quality of life outcomes, is crucial to allow governments globally to appropriately allocate often limited healthcare resources.

**PROSPERO registration number:** CRD42022348529.

## Introduction

Every year, more than five million people die from traumatic injuries worldwide, significantly more than the number of deaths from Human Immunodeficiency Virus (HIV), tuberculosis, and malaria combined [1], and disproportionately affects low- and middle-income countries (LMICs) [2]. Patient outcomes following trauma vary, but estimates from the Global Burden of Disease data suggest that around two million lives per year could be saved if case fatality rates among seriously injured persons in LMICs were similar to those achieved in high-income countries (HICs) [3]. In addition, the burden of trauma disproportionately affects those aged less than 30 years old, suggesting that any improved morbidity outcomes may have significant socio-economic implications.

For over 15 years, trauma systems have been implemented across multiple countries around the world [4,5,6,7,8,9]. A trauma system involves optimising and linking every aspect of the trauma care pathway, from pre-hospital triage protocols to pre-defined transport links, often with inter-regional variation in their components [10]. Indeed, trauma systems should not be viewed as solely the acute hospital aspect of care, but more holistically, with all community measures and rehabilitation platforms assigned equal focus and importance [11,12]. Multiple systematic reviews have demonstrated that the implementation of a trauma system results in significant improvements to overall mortality rates in trauma patients [10,13,14], yet less is known about how they influence patient quality of life and economic parameters. Much of the published literature on the impact of trauma systems derives from HIC-obtained data, however understanding this impact is of critical importance especially to LMIC governments, when choosing how to marshal often limited healthcare resources.

Traumatic injuries are heterogeneous, with certain subtypes having specific treatment requirements. For instance, Traumatic Brain Injury (TBI) forms a major subset of overall trauma care and results in a sizeable proportion of morbidity globally, with a high burden of disability and lost economic productivity seen, particularly in LMICs [15]. TBI rates remain on the rise in LMICs, mainly in young adults involved in road-traffic accidents [16,17]; if outcomes are to be improved, then it is essential that current co-ordination of TBI care is improved, through the establishment of functional trauma networks [15]. Paediatric injury is another area of trauma care that is often neglected [18], especially in trauma systems research, however arguably results in the largest impact on economic burden and Quality-Adjusted Life Years (QALYs).

We aim to perform a systematic review to explore the impact that the implementation of a trauma system can have on morbidity, quality of life, and economic outcomes to a region, with specific planned sub-analysis for TBI and paediatric cohorts.

## Methods

This study will be reported in accordance to the standard Preferred Reporting Items for Systematic Reviews and Meta-Analyses (PRISMA) guidelines [19]. The study has been registered with PROSPERO (https://www.crd.york.ac.uk/prospero/) prior to the initial literature search being undertaken. The full planned search strategy used is provided in the Supplementary Material, which have been developed in close concordance with a medical librarian.

### Eligibility Criteria

We will include any study that assesses the impact that implementation of an organised trauma system has on morbidity, quality of life, or economic outcomes. An organised trauma system will be defined as any “pre-planned approach to the provision of the spectrum of trauma services” [20]. Articles with a particular focus on trauma systems from particular clinical phenotypes, such as neurotrauma, paediatric trauma, or burns, will be included, however those assessing the impact of just a sub-component of a trauma system will not be included.

Included studies must compare outcomes against an appropriate comparator group, namely a health service that does not have an explicit organised trauma system in place; this can be either from pre-implementation data within the same region or against a comparable region without an organised trauma system in place. Due to significant developments in trauma care in recent years [21], only studies published since the year 2000 will be included. We will attempt to include studies in all languages, using university library language services for full text translations if required.

We will include any comparator study, including cohort, case-control, and randomised controlled studies, both retrospective or prospective in nature. We will include studies conducted from any region in the world and include all ages of patients (including paediatrics). Case studies or case series (<10 patients), review pieces, or editorials will be excluded, as will studies on the implementation of a specific technique or equipment involved in a trauma system (i.e. not a system intervention).

### Outcomes

We will include studies that include any morbidity outcomes (e.g. extended Glasgow Outcome Score, eGOS), health-related quality of life (HRQL) measures (e.g. Short Form 36 Questionnaire, SF-36), or health economic assessments (e.g. EuroQol Questionnaire, EQ5D) as their primary or secondary outcomes. We predict a high heterogeneity in utilised outcome measures, therefore will keep our inclusion criteria broad; certainly for the economic measures, this may include either individual or societal outcome measures, both quantitative or qualitative.

We will define a HRQL outcome as any that measures “an individual’s or group’s perceived physical and mental health over time” [22] and we will define a disability outcome as any that measures “impairments, activity limitations, and participation restrictions” [23]. Cost-effectiveness outcomes will be any factor outlined in the WHO Manual for estimating the economic costs of injuries secondary to interpersonal and self-directed violence [24]; these include independent living, loss of livelihood, return to work, out-of-pocket expenses, indirect financial costs, health expenditures, cost analysis, and socioeconomic factors.

### Data Analysis

Following the initial literature search, all studies will be reviewed independently by two separate authors using systematic review software, Rayyan [25]. Studies will initially be screened by title, then by abstract, and then full articles. Any disagreements will be resolved by a third author. A risk of bias assessment will be performed by two separate independent reviewers, using the Risk of Bias in Non-randomized Studies of Interventions (ROBINS-I) tool or the Cochrane Risk of Bias tool for randomised trials; publication bias will be assessed visually using a funnel plot where feasible.

Data analysis following extraction will be a descriptive summary of all included studies, due to the likely heterogenous nature of the studies identified, both in their methodology and outcomes. The data extracted from each included paper will include any details regarding the trauma system and its key components, the year of implementation and the region it serves, and any qualitative or quantitative data available on morbidity, quality of life, or economic outcomes. The narrative analysis will outline both the similarities and the differences between the included studies and detail any unique aspects of individual studies identified. We will conduct our narrative synthesis in accordance to the SWiM guidelines for quantitative data [26] and the ENTREQ guidelines for qualitative data [27].

We will consider further statistical analysis if sufficient quantitative data are present, however this is likely to be limited by the heterogeneity of included studies. Specific sub-analysis on Traumatic Brain Injury (TBI) patients and paediatric patients will be performed if sufficient data is available within these cohorts. An initial scoping of the available literature points to a highly heterogeneous body of work, preventing the prospective identification of an appropriate quantitative meta-analysis methodology.

## Discussion

We present a study protocol for a systematic review that will assess the impact on morbidity, quality of life, and economic outcomes following the implementation of a trauma system. Previous reviews have shown the significant improvements that can be achieved to mortality outcomes with the implementation of an organised trauma system [10,13,14], however the wider impact they can have on morbidity, quality of life, and health economic outcomes is less clear.

Rather than representing treatment received solely within a healthcare facility, trauma systems in fact comprise of multiple components, from transport and triage through to recovery and rehabilitation. Indeed, trauma pathways can be conceived of as a circular journey that begins and ends within the community, and in which public and population health measures are as important as those within the acute care system [28]. Implementing and strengthening a trauma system as a whole should not only improve trauma patient healthcare outcomes, but can have positive effects on other areas of a healthcare system, from improved infrastructure to enhanced coordination efforts [29]. In order for policy makers to realise the impact that implementation of a trauma system could have on their healthcare system, exploring all of the available evidence is essential.

Having focused sub-analyses looking at TBI care and paediatric trauma respectively in this review focuses on two areas of trauma that have a large influence on healthcare economics. In LMIC, it is known that the majority of TBI cases occur in young adults, therefore combined with paediatric trauma these two areas produce potentially the greatest loss to Quality-Adjusted Life Years (QALYs) and the greatest impact on lost economic productivity in a region of any trauma subtype. Time-critical transfer of TBI patients is known to produce optimal care, however as highlighted in The Lancet Neurology Commission on TBI [15], this can only occur through established trauma networks. Providing the core data to governments and policymakers to ensure these important areas are addressed can then allow for appropriate trauma systems to be designed and implemented.

Our proposed review does come with several limitations. We expect a high level of heterogeneity among the morbidity, quality of life, and health economic outcome measures reported in the included studies, which will likely preclude any quantifiable analysis. Indeed, this has led to the aims of our study being broad and wide-reaching, to ensure we are able to capture and present the maximal data available on this topic. We are also excluding any studies that assess only a sub-component of a trauma system, however this is to ensure we examine the true effect that a full trauma care pathway can infer on a region, not just focused aspects.

Understanding the impact trauma systems can have on wider parameters, such as economic and quality of life outcomes, remains crucial to allow governments in LMICs to appropriately allocate what are often limited healthcare resources. Furthermore, humanitarian organisations, such as the World Health Organisation, require high-quality evidence in economic and morbidity data, not purely mortality, if they are to strengthen their arguments to convince governments to invest in improved trauma care.

## Additional File

The additional file for this article can be found as follows:

10.29337/ijsp.187.s1 Supplemental Material. Databases Involved and Search Strategy.
