# Supplementary material for: The Impact of Trauma System Implementation on Patient Quality of Life and Economic Burden: A Systematic Review Study Protocol
Source: Int J Surg Protoc. 2023 Feb 9;27(1):84–9. doi: 10.29337/ijsp.187 (PMC9983497; doi:10.29337/ijsp.187)
Supplement: Supplemental Material. — Databases Involved and Search Strategy. [file ijsp-27-1-187-s1.pdf]

Supplementary Material

Databases Involved

- Medline via Ovid
- Embase via Ovid
- Web of Science Core Collection
- PsycInfo via Ebsco
- Global Health via Ebsco
- SciELO via Web of Science
- WHO Global Index Medicus: African Index Medicus, Index Medicus for Eastern Mediterranean Region, Index Medicus for South-East Asian Region, Latin America and the Caribbean Literature on Health Sciences (Lilacs), and Western Pacific Region Index Medicus

Search Strategy

Example from Medline search

1 ("quality of life" or qualy or "quality adjusted life" or "burden of disease" or "disability adjusted life year" or "disability-adjusted life year" or DALY).ti,ab.

2 ("economic burden" or "burden on the state" or "economic productivity" or "earning potential" or "cost of injur*" or employment or employed or income or welfare or poverty or socioeconomic or unemployed or unemployment or job loss or redundan* or value of life or life valuation*).ti,ab.

3 (Morbidity or co-morbid* or comorbid*).ti,ab.

4 (sf36 or "sf 36" or sf6 or "sf 6" or sf12 or "sf 12" or "sf 16" or sf16 or sf20 or "sf 20").ti,ab.

5 ("burden of disease" or wellbeing or well being or health status or health state* or health level* or "functional outcome*" or "recovery period").ti,ab.

6 (long term outcome* or longterm outcome* or (duration adj3 recovery)).ti,ab.

7 exp "Cost of Illness"/ or exp "Quality of Life"/ or exp "Value of Life"/ or quality-adjusted life years/ or exp health status/ or employment/ or unemployment/ or social welfare/ or poverty/ or social class/ or morbidity/

8 or/1-7

9 (trauma adj (service* or centre* or center* or system* or pathway*)).ti,ab.

10 exp Trauma Centers/

11 or/9-10

12 8 and 11

13 limit 12 to yr="2000 -Current"
